# Gender Differences in the Presentation of Observable Risk Indicators of Problem Gambling

**DOI:** 10.1007/s10899-017-9691-5

**Published:** 2017-05-11

**Authors:** Paul Delfabbro, Anna Thomas, Andrew Armstrong

**Affiliations:** 1 School of Psychology, University of Adelaide, Adelaide, Australia; 2 Australian Gambling Research Centre, Australian Institute of Family Studies, Melbourne, Australia; 3 Faculty of Health Arts, and Design, Brain and Psychological Research Centre, Swinburne University of Technology, Hawthorn, Australia

**Keywords:** Problem gambling, Responsible service of gambling, Harm minimisation, Gender, EGM, Identification, Customer behaviour, Staff training

## Abstract

In many countries where gambling is legalised, there has been a strong public policy focus on the need for strategies to reduce gambling related harm. These have often included policies requiring staff in gambling venues to identify and/or assist people who might be experiencing gambling-related harm. To facilitate this process, researchers have developed visible behavioural indicators that might be used to profile potentially problematic gambling. Few of these studies have, however, examined whether such indicators or ‘warning signs’ might differ between men and women. In this study, we describe the results of an analysis of data drawn from 1185 fortnightly gamblers that included 338 problem gamblers as classified by the Problem Gambling Severity Index. Indicators of problem gambling were similar between males and females with a few key exceptions. Indicators reflecting emotional distress were more commonly reported by females with gambling problems, whereas problem gambling males were more likely to display aggressive behaviour towards gambling devices and others in the venue. Amongst males, signs of emotional distress as well as attempts to conceal their presence in venues from others most strongly differentiated between problem and non-problem gamblers. Amongst females, signs of anger, a decline in grooming and those attempts to access credit were the most distinguishing indicators. These findings have implications for the refinement of identification policies and practices.

## Introduction

In many countries where gambling is legalised, there has been an increasing legislative focus on the development of policies that might reduce or minimise the harms associated with problem gambling. Usually grounded in public health frameworks, these policies require gambling operators to implement practices that impose a duty of care towards their patrons (Hancock et al. 2008; Korn and Shaffer 1999; Productivity Commission 2010). For example, information concerning the risks of gambling and available help-services often has to be promoted in venues; staff are required to assist patrons find assistance when asked; and, staff are usually required to undertake mandatory training that makes them aware of their obligations towards patrons and how to assist them. A component of this training is often the requirement to intervene and assist when patrons display behaviours indicating that they might be experiencing problems with their gambling.

The ability for staff to observe signs of problem gambling in patrons has received some research support. In previous studies, it has been shown that it is possible (at least in theory) for problem gamblers to be differentiated from other gamblers in relation to a range of visible indicators (Delfabbro et al. 2007, 2016; Hafeli and Schneider 2006; Schellinck and Schrans 2004; Thomas et al. 2013). These indicators have generally related to the: intensity and frequency of gambling; behaviours indicating a loss of control over gambling (e.g., gambling at unusual times of the day); frequent visible attempts to obtain additional money to gamble; as well as unusual social and emotional behaviours (e.g., visible signs of anger, distress, anti-social behaviours).

All of these different indicators have been shown to be significantly more prevalent in people experiencing gambling problems than other gamblers (Delfabbro et al. 2007). Some indicators occur more commonly across a range of gamblers and are, therefore, less indicative on their own (e.g., indicators relating to how long people gamble and how they obtain money to gamble), whereas others (e.g., strong emotional reactions, asking to borrow money at the venue) are rare and are typically only reported by problem gamblers. Using statistical modelling it has been shown that if one can observe around five of the indicators, then the probability of a person being a problem gambler is around 90% (Delfabbro et al. 2016). Although there is limited analysis of the extent to which venue staff can actually apply these problem gambling indicators in practice or whether they result in better outcomes for problem gamblers, such work does at least provide staff with some way to narrow down the patrons who might be at greater risk or who should be treated as patrons of interest.

In much of this work, gamblers have been treated as a relatively homogenous group. However, it is known that there may be some gender variations in how problem gambling behaviours manifest depending upon the nature of the gambling activity, its context (e.g., land-based vs. online) and the characteristics of the gamblers themselves (Balodis et al. 2014; Gainsbury 2015; Thomas et al. 2009, 2011a, b). Another potential source of variation is how visible indicators might physically manifest themselves differently based on gender. Gender differences of this nature could provide insights into differences in how men and women experience problem gambling. In the context of identifying problem gamblers in venues, any differential validity in indicators as they might be applied to men and women is vital information for staff. This knowledge may, for instance, encourage staff to use different ways of approaching men and women in venues or influence the gender of the venue staff member who might be called upon to offer advice, make an approach or offer an intervention.

Gender differences in the behavioural presentation of gambling problems have generally not been evidenced. However, there is a broader literature which suggests that while males and females are more similar than different in what constitutes problem behaviour, they do differ in their gambling habits, their motivations for gambling, and how and why gambling problems develop (Delfabbro 2000; Díez et al. 2014; Di Dio and Ong 1997; Hing et al. 2014; Holdsworth et al. 2013; Loughnan et al. 1996; Merkouris et al. 2016; Thomas and Moore 2003; Scannell et al. 2000; Stark et al. 2012; Trevorrow and Moore 1998; Wong et al. 2013). Women tend to gamble on a narrower range of activities than males and are less likely to gamble on strategic games such as casino table games and sports or horse wagering. They typically start gambling later in life than males (usually when they are adults) and typically gamble with lower intensity.

The trajectory for the development of problem gambling also differs in that women tend to develop problems more quickly than men (known as telescoping), whereas men often have gambling problems that extend back into adolescence (see Merkouris et al. 2016). Important psychological gender differences have also emerged McCormick et al. (2012). As this article shows, female problem gamblers are significantly more likely than males to report a history of childhood abuse, high psychological distress, and gambling as a form of avoidant and emotion-based coping and gambling. This may partly reflect gambling choices as gambling on electronic gaming machines (EGMs), in particular, has been shown to be associated with a personal history of early trauma or abuse and other emotionally difficult life-events and this is a preferred form of gambling for females, particularly so for female problem gamblers. Men have been found to be higher on impulsivity and to report higher rates of substance and alcohol use (see Merkouris et al. 2016 for a review of gender differences).

Given these observations, the aim of the present paper was to conduct comparisons of the extent to which male and female problem gamblers report a range of potentially visible behavioural indicators of problematic gambling. These findings draw upon data previously collected by Delfabbro et al. (2007) and Thomas et al. (2013) which focused exclusively on combined samples and which did not conduct any analysis to investigate the possibility of gender differences. Inclusion of these analyses has the potential to strengthen the validity of findings by confirming that they can generally be applied to both male and female gamblers. At the same time, the research may also suggest the presence of gender differences and therefore a potential to refine the application of indicator checklists. The indicators used in the present set of analyses were clustered into several groups: duration and intensity of gambling; impaired control; getting money to gamble; social behaviours; emotional response; and irrational behaviours. Based on prior literature, it was hypothesised that female problem gamblers would be less likely than males to report high intensity gambling, but that they be more likely to display emotional distress during gambling as based on research which has reported high rates of co-morbid anxiety and depression amongst women who gamble on EGMs (McCormick et al. 2012). No other specific hypotheses were advanced for other indicators in the absence of consistent evidence to suggest the potential existence of gender differences.

## Method

### Participants

The data used in this paper were obtained from two large studies of regular gamblers (Delfabbro et al. 2007; Thomas et al. 2013). The two studies were combined because they were independent samples and to allow for greater statistical power. Although the proportion of problem gamblers was high in each separate sample, this is diminished when the sample is broken down by gender. Given no reason to suspect that there would be any difference in the pattern of gender differences in the two samples, it was felt that valid findings would still emerge from a combined analysis. The first study (in 2007) involved 680 people who reported at least fortnightly involvement with a continuous form of gambling (gaming machines, casino table games or wagering activities). The sample comprised 300 men (44.1%) and 380 women (55.9%) drawn from 3 Australian jurisdictions (South Australia, New South Wales and the Australian Capital Territory). Just under a quarter were aged 18–35 years (22.5%), 39% were aged 36–55 years and the remainder were aged over 55 years. The second or 2013 survey involved 505 regular EGM gamblers from across Australia (Thomas et al. 2013). There were 225 women ranging in age from 18 to 98 (M age = 43.61, SD = 15.71) and 280 men ranging in age from 18 to 82 (M age = 34.84, SD = 16.05). When combined, the two surveys yielded a total sample of 1185 (580 men, 605 women).

Using the Problem Gambling Severity Index (PGSI) (see details below), it was possible to identify 338 participants as scoring over the threshold for problem gambling and 839 other gamblers (scoring below the threshold). A total of 274 males (or 30.3%) and 164 females (27.3%) scored over the threshold for problem gambling. No significant association was found between gender and problem gambler status, *χ* ^2^(1, N = 1176) < 1.

Both studies received full ethics approval prior to being conducted. In 2007, participants were recruited by a professional marketing company outside a random sample of clubs and hotels in South Australia as well as by advertisements placed into community newspapers. Participants completed surveys face-to-face at venues or by returning mailed out surveys. In 2013, short advertisements were placed on Facebook and in venues. Participants were provided with a link that took them to the online study survey. In both surveys, participants were provided with a $25 to $30 honorarium, but all data were converted to a de-identified form in the final data analysis.

## Measures

### Demographics

These questions captured the participants’ gender, age, country of birth, ethnicity, state of residence, marital status and occupational status.

### Gambling Frequency

Both studies included measures of the frequency and type of gambling participated in over the previous 12 months. In each case frequency was measured on a 9-point scale where 0 = *(0 times over the past year)* and 9 = *(More than 5 times a week)*.

### Problem Gambling

The *Problem Gambling Severity Index* (PGSI; Ferris and Wynne 2001) is part of the Canadian Problem Gambling Index. This was used to assess the severity of problem gambling for this study. The PGSI consists of 9 items and captures both gambling behaviour (e.g., ‘Have you gone back another day to try to win the money you lost?’) and the adverse consequences of gambling (e.g., ‘Has your gambling caused you any health problems, including stress or anxiety?’). Items are rated by participants on a 4-point Likert scale where 0 = *(Never)* and 3 = *(Almost always)*. Scores are summed across the whole scale and range from 0 to 27. Risk levels as set by Ferris and Wynne were as follows: 0 = Non problem gambling, 1–2 = Low risk gambling, 3-7 = moderate risk gambling, 8 + = problem gambling. Research indicates the PGSI is psychometrically sound, with demonstrated high internal consistency (α = .84–92), stability (test–retest at 3–4 weeks .78), and validity with high correlations between the PGSI and other measures of problem gambling (Ferris and Wynne 2001). The Cronbach’s Alpha was over .90 in both samples.

### Visible Behaviours and Indicators

A detailed checklist of visible indicators was developed in the 2007 study based on the methodological strategies used by Schellinck and Schrans (2004) and also Hafeli and Schneider (2006). Respondents were presented with a series of statements and were asked to report how often they engaged in the particular behaviour on a verbal-numeric scale, 1 = Never (0% of the time), 2 = Rarely (Fewer than 1 in 4 times you gambled), 3 = Occasionally (25-50% of the times you gambled), 4 = Frequently (50% of time or more often), and 5 = Always (100% of the time). Indicators were divided up into categories similar to those used by Hafeli and Schneider, but the range of items was extended to include items arising from other sources, including consultations with venue workers, counselors and researchers working in the field (see Delfabbro et al. 2007 for a summary). Indicators were not specifically categorized when administered. The item list was then extended in the 2013 study (Thomas et al. 2013) to include additional items that were indicated as being potentially important in the 2007 study. The final 52 items included: 12 items related to the frequency, duration and intensity of gambling; 5 related to impaired control; 8 items captured social behaviours; 9 related to raising money or chasing behaviours; 11 related to emotional responses; and 7 related to various other behaviours including drinking alcohol while gambling, a decline in grooming/appearance, irrational attributions for losing and avoiding the cashier. As a result of the differences between the two surveys, the sample size for the analysis of some items is smaller (i.e.,. is only based on the 2013 sample where new items were introduced).

## Results

### Gender Differences in the Prevalence of Indicators

The aim of the first set of analyses was to test for gender differences in visible indicators reported by those over the threshold for problem gambling. A summary of these results is provided in Tables 1 and 2. Table 1 shows the proportion of males and females who reported these behaviours (rarely or more often). The overall pattern of results suggested that there were generally a greater number of similarities than differences. A similar proportion of male and female problem gamblers reported behaviours indicating high frequencies and intensity of gambling and there were no significant differences related to behaviours relating to obtaining funds to gamble. The pattern of timing of gambling was also generally similar (i.e., whether they gambled at the opening or closing periods of venue operation).

**Table 1:** Gender differences in the prevalence of self-reported behavioural indicators in problem gamblers

Indicators	Men n (%)	Women n (%)	Χ^2^
Frequency, intensity, duration			
Gambled every day of the week	129 (74.1)	109 (66.9)	2.14
Gamble for 3 + hours without a break of 15 min or longer	154 (88.5)	129 (78.7)	6.01*
Gamble for 5 + hours without a break of 15 min or longer	78 (66.1)	66 (79.5)	4.32*
Gambles intensely (does not react to external stimuli)	144 (82.8)	141 (86.0)	<1
Plays very fast (inserting money/pushing buttons rapidly)	157 (90.2)	143 (88.3)	<1
Bet $2.50 or more per spin most of the time	109 (89.0)	72 (87.8)	<1
Plays on quickly after wins (not listening to music or jingle)	147 (93.0)	138 (92.6)	<1
Rush from one machine to another	147 (84.5)	133 (82.1)	<1
Gamble on 2 or more machines at once	62 (59.0)	35 (48.6)	1.88
Gamble continuously	160 (92.5)	145 (88.4)	1.62
Spend more than $300 in one session of gambling	103 (87.3)	71 (85.5)	<1
Significantly change (increase) in expenditure pattern	105 (58.3)	75 (41.7)	<1
Impaired control			
Stop gambling only when the venue is closing	134 (77.0)	113 (68.9)	2.82
Gamble right through your usual lunch break or dinner time	120 (69.0)	105 (64.4)	<1
Find it difficult to stop gambling at closing time	127 (73.0)	109 (66.5)	1.71
Try obsessively to win on a particular machine	163 (93.7)	155 (94.5)	<1
Start gambling as the venue is opening	100 (57.5)	103 (62.8)	1.00
Social behaviours			
Ask venue staff to not let people know they there	47 (27.2)	37 (22.6)	<1
Have friends or relatives call or asking if you are still there	82 (47.1)	62 (37.8)	3.00
Act rudely or impolitely to venue staff	66 (38.6)	35 (22.2)	10.44***
Avoid contact, or communicate very little with anyone else	138 (79.3)	135 (82.3)	<1
Stay on to gamble while your friends leave the venue	132 (75.9)	120 (73.4)	<1
Become very angry if someone takes favourite machine/spot	118 (67.8)	112 (68.7)	<1
Brag about winning or make a big show of gambling skill	84 (71.2)	40 (48.2)	10.90***
Stand over other players while waiting for favourite machine	63 (53.4)	30 (36.1)	5.83*
Raising funds/chasing behaviour			
Get cash out (ATM/EFTPOS) on 2 + occasions in single session	158 (91.3)	149 (90.9)	<1
Ask to change large notes at venues before gambling	144 (82.8)	131 (79.9)	<1
Borrow money from other people at venues	88 (50.6)	71 (43.3)	<1
Ask for a loan or credit from venues	40 (23.0)	33 (20.1)	<1
Put large win amounts back into the machine, continue playing	162 (93.1)	155 (95.1)	<1
Leave the venue to find money to continue gambling	143 (82.2)	135 (82.3)	<1
Rummage around in your purse or wallet for additional money	104 (88.1)	74 (89.2)	<1
Run out of all money including in purse/wallet when leave	111 (94.1)	81 (97.6)	<1
Use the coin machine at least 4 times in a session	96 (56.1)	75 (43.9)	3.01
Emotional responses			
Find yourself shaking (while gambling)	110 (63.6)	96 (58.6)	<1
Sweat a lot (while gambling)	119 (68.4)	83 (50.6)	11.1***
Feel nervous/edgy (e.g., leg switching, bites lip continuously)	142 (81.6)	133 (82.1)	<1
Display your anger (e.g., swearing to yourself, grunts)	138 (75.3)	83 (51.2)	21.0***
Kick or violently strike machines with fists	78 (44.8)	38 (23.3)	17.26***
Feel very sad or depressed (after gambling)	159 (91.9)	159 (97.5)	5.26*
Cry after losing a lot of money	88 (50.6)	115 (70.1)	13.45**
Sit with your head in hands after losing	113 (65.7)	97 (59.1)	1.54
Play the machine very roughly and aggressively	77 (65.3)	31 (37.1)	15.26**
Groan repeatedly while gambling	85 (72.0)	41 (49.4)	10.67***
Feel a significant change in your mood during sessions	110 (93.2)	75 (90.4)	<1
Other behaviours			
Gamble after having drunk a lot of alcohol	124 (71.3)	78 (47.6)	19.73***
Avoid the cashier and only use cash facilities	86 (72.9)	57 (69.5)	<1
Notice decline in grooming/appearance	65 (55.1)	48 (57.8)	<1
Blame venues or machines for losing	132 (75.9)	107 (65.6)	4.26*
Complain to staff about losing	74 (42.5)	57 (34.8)	2.15
Swear at machines or venue staff because you are losing	98 (56.3)	54 (32.9)	18.67***
Compulsively rub the machine	60 (50.8)	38 (45.8)	<1

* *p* < .05, ** *p* < .01, *** *p* < .001

**Table 2:** Ratios of the percentage endorsement of indicators (problem gamblers/non-problem gamblers) for men and women separately

Indicators	Male higher	Men	Women
Frequency, intensity, duration			
Gambled every day of the week		2.21	2.62
Gamble for 3 + hours without a break of 15 min or longer		2.40	2.41
Gamble for 5 + hours without a break of 15 min or longer	X	4.18	3.11
Gambles intensely (does not react to external stimuli)		3.01	3.76
Plays very fast (inserting money/pushing buttons rapidly)		1.80	2.45
Bet $2.50 or more per spin most of the time		1.83	2.03
Plays on quickly after wins (not listening to music or jingle)		1.45	1.53
Rush from one machine to another		2.16	2.49
Gamble on 2 or more machines at once		2.31	2.37
Gamble continuously		2.32	2.82
Spend more than $300 in one session of gambling		2.30	2.89
Significantly change (increase) in expenditure pattern		1.73	2.17
Impaired control			
Stop gambling only when the venue is closing		2.52	2.46
Gamble right through your usual lunch break or dinner time		3.67	4.60
Find it difficult to stop gambling at closing time		4.01	5.19
Try obsessively to win on a particular machine		1.62	1.71
Start gambling as the venue is opening		2.67	2.64
Social behaviours			
Ask venue staff to not let people know they there	X	**12.36**	6.64
Have friends or relatives call or asking if you are still there		4.62	6.41
Act rudely or impolitely to venue staff		4.02	4.93
Avoid contact, or communicate very little with anyone else		2.33	2.74
Stay on to gamble while your friends leave the venue		2.36	2.54
Become very angry if someone takes favourite machine/spot		3.15	3.61
Brag about winning or make a big show of gambling skill		2.01	1.77
Stand over other players while waiting for favourite machine	X	4.20	2.96
Raising funds/chasing behaviour			
Get cash out (ATM/EFTPOS) on 2 + occasions in single session		1.91	2.08
Ask to change large notes at venues before gambling		1.74	2.14
Borrow money from other people at venues		4.32	5.70
Ask for a loan or credit from venues		**12.8**	**18.3**
Put large win amounts back into the machine, continue playing		1.94	2.30
Leave the venue to find money to continue gambling		3.43	4.62
Rummage around in your purse or wallet for additional money		1.78	1.80
Run out of all money including in purse/wallet when leave		1.90	2.42
Use the coin machine at least 4 times in a session		2.73	2.86
Emotional responses			
Find yourself shaking (while gambling)	X	**7.76**	**7.70**
Sweat a lot (while gambling)		6.39	**7.13**
Feel nervous/edgy (e.g., leg switching, bites lip continuously)		3.15	4.08
Display your anger (e.g., swearing to yourself, grunts)		3.49	5.07
Kick or violently strike machines with fists		6.22	7.28
Feel very sad or depressed (after gambling)		2.31	2.55
Cry after losing a lot of money		**9.73**	**9.87**
Sit with your head in hands after losing		5.13	6.03
Play the machine very roughly and aggressively	X	3.96	3.45
Groan repeatedly while gambling	X	3.44	2.29
Feel a significant change in your mood during sessions		2.13	2.17
Other behaviours			
Gamble after having drunk a lot of alcohol		1.35	1.78
Avoid the cashier and only use cash facilities		3.72	4.40
Notice decline in grooming/appearance		**8.74**	**16.1**
Blame venues or machines for losing		2.84	3.18
Complain to staff about losing		3.86	4.58
Swear at machines or venue staff because you are losing		2.51	2.89
Compulsively rub the machine	X	2.50	1.57

Top 5 ratios for each gender are given in bold

A high proportion of gamblers from both genders reported long gambling sessions. However, males were more likely to report gambling for 3 + hours without a break while female problem gamblers were more likely to report gambling for 5 h or more.

There were clear gender differences in social and emotional behaviours. Male problem gamblers were significantly more likely to report boasting about winning to others and swearing at or being rude towards staff. Female problem gamblers were slightly more likely to report sadness but much more likely to report crying. Men were much more likely to report aggressive behaviours such as being angry, hitting the machines, and playing aggressively. Male problem gamblers were also more likely to report groaning aloud or blaming the venue/machine when they lost, and drinking while gambling.

### Differentiation of Problem Gamblers Versus Non-Problem Gamblers: Gender Differences

A second analysis compared the prevalence of each indicator (PG/NPG) for each gender (Table 2). These ratios indicate the relative usefulness of the indicator in relation to its ability to distinguish between problem and non-problem players (higher ratios indicating a greater difference). The pattern of analysis showed that the extent to which each checklist behaviour distinguished problem gamblers from other gamblers was generally the same for men and women. That is, the behaviours that distinguished male problem gamblers from other male gamblers were highly similar to those that distinguished female problem gamblers from other female gamblers. There were no indicators where the likelihood of being a problem gambler was low for one gender (e.g. OR < 3) but high for the other (e.g. OR > 6).

Behaviours which were most likely to be indicative of gambling problems for both men and women included the prevalence of emotional and social behaviours including concealing presence in venues, asking for a loan, anger, shaking, sweating or crying, and a decline in physical grooming. Interestingly, the few instances where differences between the genders in the pattern of relationships was of note were amongst three of the most indicative problem gambling behaviours for both genders. While attempts to conceal one’s presence at a venue were highly indicative of problem gambling for both genders, this was especially so for men. For women, asking a venue for a loan or credit, or a noticeable decline in personal grooming, were especially indicative.

## Discussion

The principal aim of this paper was to refine existing analysis of gambling indicators by examining whether there are likely to be gender differences in the visible presentation of problem gambling symptoms in venue environments. Overall, the findings showed that there are likely to be more similarities between male and female problem gamblers than differences. The interpretation of the results does, however, require some distinction to be drawn between the prevalence of indicators in general as opposed to the relative probabilities of different types of gambler endorsing certain indicators in male or female samples. In relation to the *prevalence* of indicators, the results revealed several differences. Males were generally more likely than females to report anger and frustration when gambling including a greater likelihood of kicking or striking gaming machines, engaging in territorial stand-over tactics to scare customers away from EGMs they have claimed, or acting impolitely towards staff in the venue. Such findings are generally consistent with a broader psychological and sociological literature that men tend to generally display more overt or physical aggression in frustrating situations than women (Cox et al. 2003; Thomas 1993). The findings may also reflect the finding from several studies that males tend to place a greater emphasis on applying skill when gambling, trying to beat the odds or the machine (Delfabbro 2000). Such beliefs may lead to strong expectations of winning and therefore stronger feelings of being cheated or frustrated when outcomes do not turn out as expected.

Women, on the other hand, are much more likely to display visible signs of distress such as crying or other signs of sadness or depressive feelings when they are losing. The more prevalent reports of strong emotional reactions (crying and visible sadness) in female problem gamblers is consistent with research which shows that women are more likely to use EGMs as a form of emotion-based and avoidant coping that serves to temporarily negate negative affect often arising from an extended history of emotionally distressing experiences and life-events (McCormick et al. 2012; Thomas and Moore 2003). Women who gamble on EGMs are considered to be group that corresponds very closely with what Blaszcynski and Nower (2002) have classified as the ‘vulnerability’ pathway of problem gambling. Anxiety and depression are common co-morbid presentations in clinical or tertiary settings, so it is possible that some of these symptoms are emerging in venue environments, particularly when women are confronted by negative gambling events such as heavy losses. An alternative, or possibly additional, explanation is that it is more normatively acceptable for women to display visible signs of distress in public, and for men to show signs of anger (e.g., Plant et al. 2000).

Despite these gender differences in the prevalence of reported behaviours, it did not always follow that the same indicators were as useful for *differentiating* categories of gambler for men and women separately. For example, as indicated in Table 2, the ratios for most behaviours (including aggressive behaviours) were often higher for women experiencing problems compared to other women. In other words, although there may be some behaviours which are more present in men, they are not necessarily any more useful in differentiating male problematic gamblers from other males. A similar issue relates to emotional behaviours; while such behaviours are more commonly reported by women, they are not necessarily any more useful in identifying female problem gamblers from other women who gamble regularly. Male problem gamblers also tend to display these behaviours more commonly than lower risk gamblers, but at a lower overall prevalence. Instead, a more careful inspection of the results showed that the indicators that differentiated male problem gamblers from other groups were those relating to concealment whereas for women, a decline in grooming or appearance or asking for loans appeared to be particularly indicative of problems.

The practical implication of these findings for staff is that the signs of problematic gambling are likely to be similar for males and females, but that signs of distress are likely to be more commonly seen indicators for female patrons while signs of anger may be more likely to be observed for males. Further, it would be important for staff to realise that unusual behaviours such as observations of aggression towards machines, attempts to borrow money or ask for credit or a decline in grooming in female patrons are definite ‘red flags’ and similarly observing clear and visible signs of distress in male patrons or attempts to conceal their presence in venues from family and friends would be particular indicators of which staff should take note. The results also more broadly show that the behaviour of female problem gamblers is generally more differentiated from other lower risk gamblers (as evidenced by the greater number of higher ratios in Table 2). This suggests that it may be easier to detect variations in behaviour for female gamblers more easily than for males. A range of behaviours appear to be less normative in lower risk female gamblers so that it may be more common to discern variations in behaviour, but more difficult to do so for males. In effect, staff may need to spend more time watching potential male problem gamblers before they can be confident that they are displaying behaviour that is different from other male gamblers.

### Limitations and Future Directions

Despite these potentially useful applications, there are several conceptual and methodological limitations that need to be taken into account when interpreting these findings. First, at a conceptual or public policy level, it is important to acknowledge that the use of behavioural indicators in venues is essentially a reactive strategy rather than one that prevents gambling-related harm. In other words, rather than looking to find ways to prevent harm, they are predicted on the assumption that potentially problematic behaviours and harms have already developed. In effect, a person has to be displaying signs of harm to be identified as a problem gambler. For this reason, it is probably difficult to argue that venue staff training into the nature of indicators, of itself, can create a ‘responsible gambling’ environment or fulfil a duty of care to prevent the harms associated with gambling. Instead, such policies are best seen as a potential form of harm reduction that may, on some occasions, lead to problem gamblers seeking assistance to deal with their problems.

There are also several methodological limitations that need to be considered. First, the results are based on a self-report methodology, so that it is possible that people over or under-report the actual behaviours which they display when they gamble in venues. There may also be gender differences in awareness of, or willingness to report, particular behaviours. Second, the research uses behavioural observations from only a single point of reference rather than the behavioural observations of a third party. Third, analyses are also confined to the comparison of problem and other gamblers, so that further analyses could potentially be undertaken to examine whether there are indicators that allow more refined differentiation of different levels of gambling risk. For example, are there differences between male and female problem and moderate risk gamblers and/or between male and female moderate and low risk gamblers? Such comparisons may be potentially useful in that an understanding of how indicators relate to the gambling risk continuum may make them more useful for the early identification of gamblers at risk and well as greater insights into what behaviours are typical of regular gambling that does not appear to be related to harmful consequences. Fourth, it is important to recognise that this was a convenience sample rather than one drawn from the general population and which included an incentive to participate in the research. As a result, it is unclear whether the findings can be generalised to all gamblers in the general population or only the sorts of people who are willing to participate in research projects. In general population surveys in Australia, the prevalence of problem gambling is usually found to be higher in men than in women, a characteristic that was not true of the samples used in the present analyses.

Moreover, as we have noted previously (Delfabbro et al. 2016), the application of these indicators to venue environments poses a number of challenges. Although our findings suggest that it is theoretically possible to differentiate different types of gamblers, venue staff need to be able to observe these behaviours and accumulate information in a way that allows confidence in identification. It may be that in less well-resourced venues (particularly hotels and clubs with EGMs), venue staff may not be well trained and/or may not spend large amounts of time on the gaming floor (Delfabbro et al. 2007; Thomas et al. 2013). This may mean that they are less likely to obtain sufficient information about players or to correctly interpret it. Another challenge which has been previously documented in related qualitative projects (Thomas et al. 2013) is that venue staff are generally reluctant to approach patrons when they observe signs of gambling problems and fear such approaches will lead to negative responses from gamblers and/or gamblers going to other venues and becoming even more furtive in their behaviour. Our findings here suggest that possible gender differences in behaviour may potentially influence some of these challenges. If males are generally more aggressive towards staff, whereas women are generally display more emotional responses, this may make it more difficult for staff to approach males to offer assistance. It may also imply that venues may need to examine the age and gender profile of staff members so that they have the capability to deal with patrons with different characteristics. It may be, for example, having very young female staff approach older male gamblers is not appropriate or helpful and that more mature and better trained staff with experience in anger management and ‘complex customers’ will be needed to assist angry, aggressive or emotionally distraught patrons in an effective and empathic manner.

One way such issues can be addressed is to enhance regulations to provide for adequate training of staff as well as unambiguous expectations regarding staff actions including consequences for non-compliance. Staff training should aim to increase their ability to (a) identify behavioural indicators, (b) interpret the totality of behaviour and the context in which it occurs and, (c) be confident to use this information in their interactions with patrons. Some of this training might include some focus on gender differences and diversity in gamblers. In line with these suggestions, future research in this area could then examine whether more enhanced training relating to gender differences in gambling behaviour as well as the visible indicators of problem gambling might enhance the ability of staff to deal more effectively with clients who appear to be experiencing gambling-related harm in venues.
